# Pneumothorax presenting as epigastric pain

**DOI:** 10.1002/jgf2.447

**Published:** 2021-05-05

**Authors:** Junki Mizumoto

**Affiliations:** ^1^ Department of Medical Education Studies Graduate School of Medicine International Research Center for Medical Education The University of Tokyo Tokyo Japan

**Keywords:** chronic obstructive pulmonary disease, emergency medicine, epigastric pain, pneumothorax, respiratory disease

## Abstract

A male patient who smoked heavily complained of severe epigastric pain. He also had mild chest pain for the last 4 weeks. Imaging tests revealed pneumothorax secondary to cystic changes probably as a result of chronic obstructive pulmonary disease. Pneumothorax, particularly slowly progressive one, may present as abdominal pain. In addition to cardiovascular and gastrointestinal diseases, pneumothorax should be considered as a differentiasl diagnosis of epigastric pain with preceding pain or other symptoms in a heavy smoker.

## Graphical abstract

A male patient complained of severe epigastric pain. The diagnosis of pneumothorax was made. Pneumothorax, particularly slowly progressive one, may present as epigastric pain.

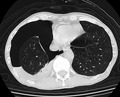

## 1 CASE PRESENTATION

A 64‐year‐old Japanese man presented to our hospital because of severe epigastric pain. Four weeks previously, the patient had developed mild right chest pain. The pain was neither alleviated nor aggravated. He had tolerated the pain and did not see a doctor because of his job. The night before presentation, the patient developed severe epigastric pain acutely, and he could not sleep at all. He had no particular medical history with the exception of heavy smoking. He had smoked at least 20 cigarettes per day for about 40 years. However, he had quit smoking 3 weeks previously because of the chest pain.

**FIGURE 1:**
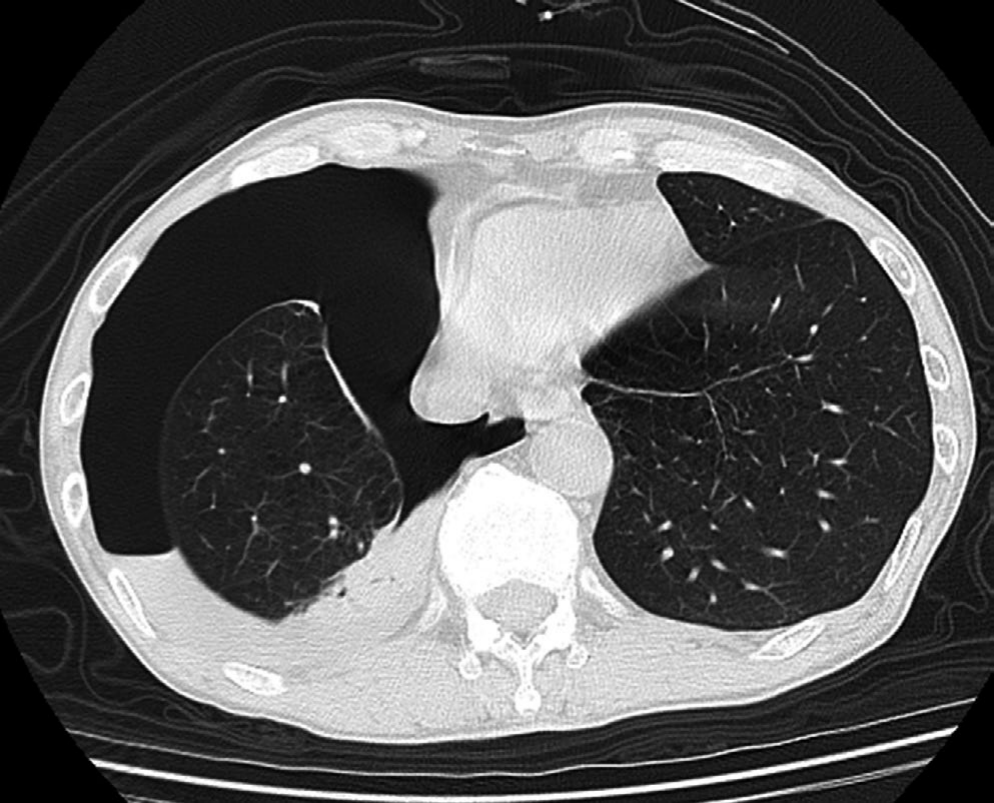
Right pneumothorax revealed by chest computed tomography

On physical examination, the patient thrashed around on the bed with forward bending because of severe epigastric pain. His vital signs were as follows: body temperature of 36.5ºC, heart rate of 116 beats/minute, blood pressure of 144/46 mmHg, and oxygen saturation of 94% on room air. Lung sounds could not be assessed because of his groaning. His abdomen was tense, and no other examinations could be performed. A computed tomography scan revealed a collapsed right lung with multiple emphysematous cysts and bullous changes (Figure 1). No abnormalities were found in the abdomen. The diagnosis of pneumothorax secondary to cystic changes probably as a result of chronic obstructive pulmonary disease (COPD) was made. The patient was admitted to the hospital and was treated conservatively. The pain gradually diminished and a computed tomography scan taken two days later revealed an expanded right lung. The patient was discharged home. On the day of follow‐up visit (1 week after the discharge), the patient did not complain of any symptoms.

## 2 DISCUSSION

Pneumothorax typically presents as pleural chest pain and dyspnea.^1^ However, some patients with pneumothorax develop abdominal pain. Although its etiology is unclear, there are three main hypotheses. One is that the diaphragm on the affected side is depressed because of tension pneumothorax.^2^ Another is that a small amount of pleural effusion causes upper quadrant pain on the affected side.^3^ The third hypothesis is that a highly collapsed lung pulls the pulmonary ligament upward, interfering with the diaphragm on the affected side.^1^ The final hypothesis is based on slowly progressive pneumothorax in heavily smoking patients, and it seemed most likely in this case.

Chronic obstructive pulmonary disease is one of the most common underlying pulmonary diseases in patients with pneumothorax, especially those aged >55 years.^4^ Lungs affected by COPD may lose elasticity because of pleural adhesions and diffuse bullae.^4^ This might have led to the slow progression of pneumothorax in the present case. That is, patients with pneumothorax secondary to COPD may develop epigastric pain rather than primary spontaneous pneumothorax.

In summary, pneumothorax may present as epigastric pain. Slowly progressive pneumothorax, such as pneumothorax secondary to COPD, and long‐term tolerance of pain may be risk factors for the development of epigastric pain. In addition to cardiovascular and gastrointestinal diseases, pneumothorax should be considered as a differential diagnosis of epigastric pain with preceding pain or other symptoms in a heavy smoker.

## CONFLICT OF INTEREST

The authors have stated explicitly that there are no conflicts of interest in connection with this article.

## INFORMED CONSENT

Informed written consent was obtained from the patient for publication of this report and any accompanying images.
